# Evidence based practice beliefs and implementation among nurses: a cross-sectional study

**DOI:** 10.1186/1472-6955-13-8

**Published:** 2014-03-25

**Authors:** Kjersti Stokke, Nina R Olsen, Birgitte Espehaug, Monica W Nortvedt

**Affiliations:** 1 Department of Oncology, Division of Cancer Medicine, Surgery and Transplantation, Oslo University Hospital, Postbox 4953- Nydalen, Oslo N-0424, Norway; 2 Centre for Evidence-Based Practice, Faculty of Health and Social Sciences, Bergen University College, Postbox 7030, Bergen N-5020, Norway

**Keywords:** Evidence-based practice, Beliefs, Behaviour, Implementation, Nurses, Survey

## Abstract

**Background:**

Having a positive attitude towards evidence-based practice and being able to see the value of evidence-based practice for patients have been reported as important for the implementation of evidence-based practice among nurses.

The aim of this study was to map self-reported beliefs towards EBP and EBP implementation among nurses, and to investigate whether there was a positive correlation between EBP beliefs and EBP implementation.

**Method:**

We carried out a cross-sectional study among 356 nurses at a specialist hospital for the treatment of cancer in Norway. The Norwegian translations of the Evidence-based Practice Belief Scale and the Evidence-based Practice Implementation Scale were used.

**Results:**

In total, 185 nurses participated in the study (response rate 52%). The results showed that nurses were positive towards evidence-based practice, but only practised it to a small extent. There was a positive correlation (r) between beliefs towards evidence-based practice and implementation of evidence-based practice (r = 0.59, p = 0.001).

There was a statistical significant positive, but moderate correlation between all the four subscales of the EBP Beliefs Scale (beliefs related to: 1) knowledge, 2) resources, 3) the value of EBP and 4) difficulty and time) and the EBP Implementation Scale, with the highest correlation observed for beliefs related to knowledge (r = 0.38, p < .0001). Participants who had learned about evidence-based practice had significantly higher scores on the Evidence-based Practice Belief Scale than participants who were unfamiliar with evidence-based practice. Those involved in evidence-based practice working groups also reported significantly higher scores on the Evidence-based Practice Belief Scale than participants not involved in these groups.

**Conclusion:**

This study shows that nurses have a positive attitude towards evidence-based practice, but practise it to a lesser extent. There was a positive correlation between beliefs about evidence-based practice and implementation of evidence-based practice. Beliefs related to knowledge appear to have the greatest effect on implementation of evidence-based practice. Having knowledge and taking part in evidence-based practice working groups seem important.

## Background

Internationally, evidence-based practice (EBP) has been a priority for many years. Both the World Health Organization and the European Commission emphasize that health and social services should be based on the best research evidence [1]. EBP is an approach that requires that decisions about health care should be based on the best available, current, valid and relevant evidence [2,3]. In addition, evidence-based decisions should be made by those receiving care, informed by the tacit and explicit knowledge of those providing care, within the context of available resources [3]. EBP involves the following steps: asking clinical questions, searching for and collecting the most relevant best evidence, critically appraising the evidence, integrating the evidence with one’s clinical expertise, patient preferences and values, and evaluating outcomes of the practice decision or change made on the evidence [4]. EBP implementation involves use of the EBP steps and strategies that promote integration of best available evidence with practitioner expertise and other recourses [5].

Finding, appraising, applying and evaluating research evidence are essential components of EBP. However, earlier studies show that nurses seldom incorporate research findings into their practice, and they tend to use knowledge derived from experience and social interactions [6-10]. These results concur with findings from surveys among Norwegian nurses [11,12]. Results from these studies show that nurse practitioners rarely use research and rely on other sources of information such as: their own and their colleagues’ practical knowledge, knowledge gained from their nursing education, nursing literature and guidance from experts [11,12].

Recent studies from various countries have reported that nurses use EBP to a limited extent [13-16]. Well documented barriers, such as lack of time to read literature and lack of authority to change practice, have repeatedly been found to hinder use of EBP among nurses [14,17,18]. Organizational barriers include lack of staff experienced in EBP, supportive leadership and lack of resources [18,19]. The many identified barriers towards EBP are not surprising considering that EBP is a process that is far from straightforward and does not follow a prescribed, logical and linear path, but is both challenging and complex [20,21].

Despite these barriers, nurses generally report positive attitudes and beliefs towards EBP and they recognize the importance of EBP for quality of care – this is independent of workplace, role, or nationality [22-26]. Findings from previous studies indicate that nurses’ attitudes and beliefs are associated with the extent to which EBP is implemented [16,27-31]. Consequently, attitudes and beliefs can potentially predict future behaviour [32].

EBP is rather a new concept in Norway and little is known about EBP beliefs and use of EBP among Norwegian nurses. In this study, we aim to explore EBP beliefs and EBP implementation among nurses in a university hospital setting, and to investigate whether there was a positive correlation between EBP beliefs and EBP implementation.

## Methods

### Participants

A descriptive comparative study design was used with a cross-sectional sampling among nurses at a specialist university hospital for the treatment of cancer. The hospital aims to implement EBP in nursing care.

The Norwegian nursing education is a 3-years bachelor program (180 ECTS) [33]. Further in the article we refer to them as registered nurses (RN). Nurses have the opportunity to conduct specialization after having gained some clinical experience for some years. Education which provides specialist expertise in nursing is done by universities and university colleges and leads in some cases to a master’s degree. The program takes from 1 to 2 years fulltime (referred as specialist nurses) [33]. We also included the nurses that were not always directly involved in patient care, such as senior charge nurses and professional development nurses at the hospital units, as support from nursing administrators and leaders, is seen as a key element to promote EBP [34].

Participants were nurses who were at work at the hospital between September 20^th^ and December 6^th^ 2010 (n = 356). One of the questionnaires asked about EBP activities done during the past 8 weeks. Exclusion criteria were, therefore, nurses who had not been at work during the previous 8 weeks (n = 2).

### Measures

We used the Norwegian translations (unpublished) of the EBP Beliefs Scale and the EBP Implementation Scale developed by Melnyk and Fineout–Overholt [35]. The EBP Beliefs Scale and the EBP Implementation Scale have been used and tested for reliability and validity in several studies among nurses [5,35-44]. In addition, demographic data were collected related to: age, seniority, job position, percentage of full-time position, education, and participation in evidence-based workgroups. We also asked if the nurses had any previous knowledge about EBP.

To find out whether respondents were different from non-respondents, we collected background information on age and continuing education for all the nurses at the hospital.

The EBP Beliefs Scale consists of 16 statements that allow measurement of an individual’s beliefs about the value of EBP and their ability to implement it [5]. In the EBP Beliefs Scale respondents are asked to score the level to which they agree or disagree with the 16 statements by answering on a 5 point Likert–scale that goes from strongly disagree (1) to strongly agree (5). Examples of statements are: *“I believe the care that I deliver is evidence-based”, “I believe that EBP results in the best clinical care for patients”.* The scoring on the 16 questions is added up to a minimum of 16 points and a maximum of 80. There are two reverse-scored items. Once reversed, all items are added to give a total score. Higher scores reflect more positive beliefs about EBP.

To study nurses’ beliefs about EBP, we used four subscales of the EBP Beliefs Scale, defined by Estrada [38]: (1) knowledge beliefs, (2) value beliefs, (3) resource beliefs, and (4) time and difficulty beliefs. Items related to knowledge beliefs consist of knowing the steps of EBP, measurement of outcomes, implementation to make practice changes and confidence (n = 5). Value items include beliefs that EBP results in the best clinical care and improves patient care (n = 5). Included in the resource items are access to the best resources and ability to overcome barriers (n = 4). Time and difficulty beliefs take in items about time to do EBP and whether nurses find EBP difficult (n = 2).

The EBP Implementation Scale consists of 18 statements that allow the participants to respond to each of the statements on a 5 point frequency scale by indicating how often in the past 8 weeks they performed the item [5]. In the EBP Implementation Scale questions are linked to actual use of EBP in professional performance measuring the essential components and steps of EBP, for example, how often have you, *“critically appraised evidence from a research study?”, or “used evidence to change my clinical practice.* The response alternatives are 0 = “0 times”, 1 = “1 – 3 times”, 2 = “4 – 6 times”, 3 = 6 – 8 times and 4, meaning “> 8 times”. Scoring consisted of summing responses to the 18 items for a total score that could range from 0 to 72. Higher total scores reflect more frequent use of EBP behaviours and skills [5]. Further, response alternatives for items related to EBP implementation were collapsed into three categories, namely “0 times”, “1 to 5 times” and “6 times or more”.

### Procedure

Each hospital unit assigned a contact person for the project. The contact person and the senior charge nurse at each unit were informed about the aim of the study. The first author then disseminated information about the survey to all nurses at the hospital a week before the data collection started. The contact persons reminded the nurses daily about the survey. In addition, they were responsible for handing out and collecting the questionnaires, including the information letters and reply envelopes. Before participation, all the nurses that participated signed a written consent form after they had received an explanation about the research, the voluntary nature of their participation, and a guarantee of anonymity. The data collection took place during a 3-week period from November 15^th^ until December 6^th^ 2010. Ethical and administrative approvals were obtained from the Health Research Ethics Board, Oslo University Hospital on October, 28^th^ 2010 (ID #25878).

### Data analysis

The IBM SPSS software Version 18.0 was used in this study.

In accordance with recommendations from Polit and Beck [45], mean substitution was used for missing data on the EBP Beliefs Scale and the EBP Implementation Scale items if less than 20% were unanswered. Internal consistency of the scales was investigated using the Cronbach’s *α.* To assess non-response bias, demographics for the entire sampled group were collected and demographics on age and level of education among respondents and non-respondents were compared.

Descriptive statistics were used to describe the background factors, as well EBP beliefs and implementation of EBP.

To investigate a possible linear relationship between attitudes and EBP beliefs and implementation we used the Pearson’s correlation coefficient (r). Partial correlation coefficients with adjustment for all background factors were also calculated.

Multiple linear regression analyses were used to determine the contribution of background variables, on EBP beliefs and implementation, respectively, while controlling for the other variables.

The level of significance was set at 0.05.

## Results

The questionnaires were distributed to 356 nurses. In total, 187 nurses returned the questionnaires and 185 of these were included (52%). Two of the respondents were excluded as they had answered less than 80% of either the EBP Beliefs or the EBP Implementation Scale questions. There were no statistically significant differences in age or level of education between respondents and non-respondents. The internal reliabilities (Cronbach's Alpha) for this study were 0.86 for the EBP Beliefs Scale and 0.85 for the EBP Implementation Scale.

Half the participants were employed as registered nurses with a Bachelor’s degree (50.8%), while 38.9% were employed as specialist nurses. Another 7.6% of the participants worked as senior charge nurses and 2.7% as professional development nurses (Table 1). The majority of participants (87%) were 80-100% full-time employees, and a small proportion worked 50% or less (6%). Almost all of the respondents (96.2%) had contact with patients in their daily work.

**Table 1:** Demographics among 185 nurses

**Variables**	Mean (SD*)	**Median**	**Min - max**
AGE	39.6 (11.6)	38	22 – 65
SENIORITY			
Total seniority (years):	13.6 (11.0)	10	0 – 42
Seniority at the specialist hospital (years):	9.6 (8.7)	7	0 – 36
	**Number**	**%**	
GENDER			
Men	10	5.4	
Women	175	94.6	
POSITION			
Reg. nurse	94	50.8	
Specialist nurse	72	38.9	
Prof. development nurse	5	2.7	
Senior charge nurse	14	7.6	
PERCENTAGE OF FULL TIME			
100%	122	65.9	
80 – 95%	39	21.1	
55 – 70%	13	7.0	
50 - 20%	11	6.0	
PATIENT CONTACT	178	96.2	
HIGHEST LEVEL OF EDUCATION			
Basic training	93	50.3	
Further training	86	46.5	
Masters degree	6	3.2	
Doctoral degree	0		

*SD = Standard deviation.

The majority of nurses (82.2%) had learned about EBP, and 69.7% of these had learned about EBP through work, 25.7% in their basic nurse education and 8.5% in their post-graduate education (Table 2). A total of 10.8% of the nurses said they took part in EBP working groups, where they either developed evidence-based guidelines, protocols or patient information or wrote scientific articles using EBP.

**Table 2:** Knowledge about EBP and participation in EBP networks among 185 nurses

		**Number (%)**
**Knowledge about EBP**	Have previous knowledge	152 (82.2)
Learned about EBP through*:	- Job	106 (69.7)
	- Basic training	39 (25.7)
	- EBP Post graduated education	13 (8.5)
	- Course	3 (2.0)
	- Other	25 (16.4)
**Participation in EBP working group**	Participate in EBP working group	21 (10.8)
Type of EBP working group*	Professional procedures	15 (8.1)
	Writing articles	8 (4.3)

*Overlap/possibilities to cross several choices in the questionnaire.

### Beliefs towards EBP

For the EBP Beliefs Scale, the average total score was 42.0 (possible scores 16 – 80) (standard deviation (SD) = 6.8, range = 20 – 70). The distribution of answers within the four subscales of the EBP Beliefs Scale is presented in Table 3. The majority of the respondents (71.8%) scored highest in the subscale «Beliefs related to the value of EBP». A total of 86% of the respondents agreed or strongly agreed with the statement that evidence-based guidelines can improve clinical practice. Further, 79% agreed or strongly agreed that critically appraising evidence is an important step in the EBP process and 78% agreed or strongly agreed that EBP resulted in the best clinical care for patients (Table 3). Only a few participants strongly agreed or agreed on the statements about having knowledge about implementing EBP sufficient enough to make practice changes (12%), measuring outcomes of clinical practice (13.5%), and whether they believe they can access the best resources in order to implement EBP (17.0%).

**Table 3:** Percentages who agree or strongly agree with the individual statements and subscales in the EBP Belief Scale among 185 nurses

**Subscales**	**Strongly agree/agree%**
**A) Beliefs related to knowledge:**	**23.7**
2. I am clear about the steps of EBP (36.2%).	
3. I am sure that I can implement EBP (34.0%).	
10. I am sure about how to measure the outcomes of clinical care (13.5%).	
14. I know how to implement EBP sufficiently enough to make practice changes (12.0%).	
15. I am confident about my ability to implement EBP where I work (23.0%).	
**B) Beliefs related to the value of EBP:**	**71.8**
1. I believe that EBP results in the best clinical care for patients (77.8%).	
4. I believe that critically appraising evidence is an important step in the EBP process (79.0%).	
5. I am sure that evidence-based guidelines can improve clinical care (86.0%).	
9. I am sure that implementing EBP will improve the care that I deliver to my patients (72.0%).	
16. I believe the care that I deliver is evidence-based (44.3%).	
**C) Beliefs related to resources:**	**33.0**
6. I believe that I can search for the best evidence to answer clinical questions in a time-efficient way (31.3%).	
7. I believe that I can overcome barriers to implementing EBP (62.2%).	
8. I am sure that I can implement EBP in a time-efficient way (24.3%).	
12. I am sure that I can access the best resources in order to implement EBP (17.0%).	
**D) Beliefs to difficulty and time**	
11. I believe that EBP takes too much time. (reverse scored) (18.9%).	**22.5**
13. I believe EBP is difficult (reverse scored) (17.3%).	

In brackets after the statements are the percentages who agree/strongly agree with the statement.

### EBP implementation

**Figure 1:**
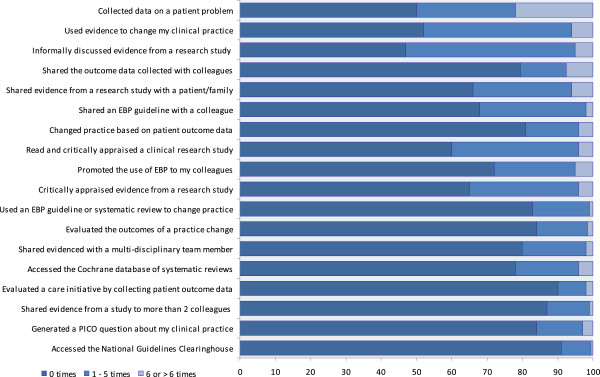
Activities carried out which are asked about in the implementation scale.

The average total score on the EBP Implementation Scale was 7.8 (possible score 0 – 72) (SD = 7.9, range = 0 – 48). Over half the participants (53%) answered that they had informally discussed a research study with a colleague more than once in the last 8 weeks. Forty per cent had read and critically appraised a clinical research study during the last 8 weeks and 34% had shared evidence from a research study with a patient/family member. Few nurses had carried out actions related to EBP (Figure 1). A total of 90% stated that they had not evaluated their own practice systematically during the last 8 weeks (Figure 1).

### Link between EBP beliefs and EBP implementation

There was a positive correlation between the EBP Beliefs Scale and the EBP Implementation Scale (r = 0.59, p = 0.001), which indicated that the stronger beliefs a nurse has in EBP the higher the nurse will report EBP implementation. This relationship was upheld after adjusting for all the registered background variables.

There was a statistical significant positive, but moderate correlation between all the four subscales of the EBP Beliefs Scale (beliefs related to: 1) knowledge, 2) resources, 3) the value of EBP and 4) difficulty and time) and the EBP Implementation Scale, with the highest correlation observed for beliefs related to knowledge (r = .38, p < .0001) (Table 4). To investigate which background variables have an independent effect on beliefs and implementation of EBP, we used multiple linear regression analysis (Table 5). The analyses showed that those who had learned about EBP (B = 2.7, 95% CI = 0.2 to 5.3) and those who took part in EBP working groups (B = 3.2, 95% CI = 0.1 to 6.3) had statistically significant higher average scores for beliefs in EBP than those who had neither learned about EBP nor worked in EBP working groups. *Registered nurses* also scored significantly lower in beliefs in EBP than more highly trained nurses (B = -4.2, 95% CI = -8.3 to -0.2). None of the background factors were associated with implementation of EBP in a statistically significant way (Table 5).

**Table 4:** Pearson correlation coefficients between the EBP Implementation Scale and the four subscales of the EBP Belief Scale estimated based on data from 185 nurses

	**Implementation**
**Attitudes related to knowledge**	0.38 (p < 0.001)
**Attitudes related to resources**	0.29 (p < 0.001)
**Attitudes related to the value of EBP**	0.29 (p < 0.001)
**Attitudes related to difficulty and time**	0.25 (p = 0.001)

**Table 5:** Factors associated with beliefs about EBP and implementation of EBP, by multivariate linear regression analyses among 185 nurses

	**BELIEFS ABOUT EBP**				**IMPLEMENTATION OF EBP**			
	**B**	**St B**	**95% CI**	**p**	**B**	**St B**	**95% CI**	**p**
AGEGROUPS								
20 – 35 years	−1.4	−0.10	(−6.0-3.3)	0.56	0.4	0.03	(−5.3-6.1)	0.89
36 – 50 years	−0.7	−0.05	(−4.0-2.7)	0.70	−0.6	−0.03	(−4.7-3.6)	0.79
> 50 years	Reference				Reference			
SENIORITY (years)	−0.1	−0.13	(−0.3-0.1)	0.36	−0.0	−0.05	(−0.2-0.2)	0.75
POSITION								
Registered Nurse (Bachelor's degree)	**−4.2**	**−0.31**	**(−8.3-0.2)**	**0.04**	−1.5	−0.10	(−6.5-3.5)	0.54
Specialist Nurse	−3.0	−0.21	(−6.3-0.3)	0.08	−0.5	−0.03	(−5.6-3.6)	0.81
Senior charge- and Prof. Development Nurse	Reference				Reference			
HIGHER LEVEL OF EDUCATION (yes vs no)	2.7	0.20	(−0.3-6.3)	0.06	2.3	0.14	(−1.15-5.8)	0.19
LEARNED ABOUT EBP (yes vs no)	**2.7**	**0.15**	**(0.2-5.3)**	**0.04**	0.2	0.01	(−3.0-3.3)	0.91
PARTICIPATION IN EBP WORKING GROUPS (yes vs no)	**3.2**	**0.15**	**(0.1-6.3)**	**0.05**	3.5	0.14	(−0.4-7.3)	0.08

Statistically significant associations (p < 0.05) are **highlighted.**

B=regression coefficient; St B=standardized regression coefficient; CI=confidence interval for B; p=p-value.

## Discussion

The results showed that nurses were positive towards EBP, but only practised EBP to a small extent. Belief in EBP was significantly higher among those who had learned about EBP and those who participated in EBP workgroups. The analysis of each subscale of the EBP Belief Scale showed that our sample, to a large degree, believe in the value of EBP. However, they have less faith in their own knowledge in relation to EBP. When it comes to beliefs related to resources for EBP the nurses scored low. Most nurses in our study “neither agree nor disagree” with the question about whether EBP is difficult and takes time. Furthermore, there was a correlation between beliefs and EBP implementation.

This study was conducted among all nurses employed at a selected hospital, regardless of their knowledge of EBP. The average total score on the EBP Implementation Scale proved to be low compared to earlier studies done among nurses, using the same scale (7.8 of a possible 0 – 72) [5,29,39-43,46]. Wallen, Mitchell, Melnyk et al. [43] found the highest average score on the EBP Implementation Scale (40.9 of possible 72). The respondents in this study participated in an EBP mentorship program (n = 54). They included nurses in management and senior positions that would lead and mentor nurses throughout the nursing department [43]. Given the high levels of competence among these participants, the high average score in the study is not surprising.

Despite the low average score on the EBP Implementation Scale in our study, we saw signs of EBP implementation. For example, half of the participants had discussed a research article informally with a colleague during the previous 8 weeks and around a third had read and critically appraised a research article and communicated evidence from a research study to a patient/relative or told a colleague about a clinical guideline. However, hardly any of the respondents (90%, n = 167) had evaluated their own practice systematically. Consequently, this is an area that needs to be focused on in the future, for example by audit and feedback. Audit and feedback is widely used as a strategy to improve professional practice either on its own or as a component of multifaceted quality improvement interventions [47].

The participants in our study had low scores on their beliefs about resources linked to EBP. In particularly, they were unsure about whether they had access to the best resources needed to apply EBP, although health workers in Norway have free internet at work and free access to scientific articles via the Norwegian Electronic Health Library [48]. Access to literature is seen as an important initiative for implementing EBP [49,50]. Several studies have pointed out that, when planning to introduce EBP, there must be opportunities to search for literature and to find systematic reviews and articles in full-text versions [50-52]. It may be that the participants in our study have a different understanding of the word “resources”, but it is also possible that they are not aware of or familiar with the Norwegian Electronic Health Library.

Most nurses in our study “neither agreed nor disagreed” with the question about whether EBP is difficult and takes time. A possible explanation may be that few nurses have been involved in defined EBP activities, and therefore they had no real concept of whether or not EBP was difficult or time-consuming. This finding differs from other studies, where nurses consistently report lack of time as a considerable barrier, and lack of time is the most frequently cited barrier to use of research [53]. Daily responsibility for many patients per nurse and for advanced care can be difficult to combine with carrying out EBP. On the other hand, the high workload and the level of responsibility within nursing strengthen the need for basing practice on the best available and updated evidence. Administrative support, proper planning and use of human resources are therefore essential to allow nurses to fit EBP into a busy schedule.

There was a positive correlation between the EBP Belief Scale and the EBP Implementation Scale, even after adjusting for background variables. This may mean that the individual’s attitude to EBP is related to the extent to which they carry out EBP. If so, future interventions should influence nurses’ perceptions of the advantages of EBP in improving clinical care and patient outcomes. This could give nurses more motivation to learn about and engage in evidence-based work.

There was a significant correlation between all four subscales of the EBP Belief Scale: (1) knowledge beliefs, (2) value beliefs, (3) resource beliefs, and (4) time and difficulty beliefs and the EBP Implementation Scale. In our study, the sample had a high level of belief in the value of EBP, but a lower level of beliefs in their own knowledge about EBP. At the same time, beliefs about their own knowledge correlated most strongly with the EBP Implementation Scale. These findings seem to be consistent with the Transtheoretical Model of Organizational Change. When knowledge about EBP is developed and conviction is strengthened, the individual will be motivated to get involved and work in an evidence-based way [41].

Earlier studies support the idea that attitudes towards and implementation of EBP can be influenced by education and competence building, accessible resources, making time available, and use of EBP mentors who support nurses in implementing EBP [22,49-52,54,55]. Thus, nurses who have knowledge and competence in EBP; access to resources; experience support; or are active in developing evidence-based guidelines and procedures have more belief in EBP.

A key question that has arisen is whether it is reasonable or achievable that every nurse should know and follow all the steps in EBP, and if it is desirable that each individual nurse is able to adjust his/her practice on the basis of valid and relevant current research. At the hospital where our study was conducted, a group of selected nurses had received training in EBP in order to become an expert resource within EBP. Educating a selected group of clinicians is in line with suggestions from several researchers who recommend that not all employees need to be trained to an expert level where they can find, evaluate, implement and generate research [55,56]. Instead, each department should have some EBP experts to engage the other staff and maintain an EBP approach.

Ciliska [57] suggests that every nurse should at least have an understanding of the purpose and process of EBP, be able to ask relevant clinical questions, and know who in their environment can assist them in answering questions. It is important to make the entire staff aware of EBP and conscious of reflection in practice, as well as learning to ask questions. It is the clinician who knows the practice area best, and who has direct patient contact. The starting point for evidence-based practice is clinical activity. Currently a group of nurses with expertise in EBP appear to be the ones who seek out whatever answers are available. These nurses are at a prime interface of recognizing clinical problems, having the skills and resources to access the research literature, critically appraising the relevant literature, and translating the findings in a way that front-line nurses can understand.

A strategy to give staff the chance to practise in an evidence-based way without having to have an in-depth competence in the entire process may be to develop procedures, best practices and guidelines that use high quality evidence. However, high quality guidelines, procedures and recommendations that include updated research results are not something that just comes to us. At the hospital where we conducted our study, it was the clinicians who identify a problem or a dilemma related to a certain procedure, patient information or nursing intervention. For this reason, experts within EBP together with interdisciplinary experts develop evidence-based guidelines, procedures and patient information in evidence-based working groups. The work was organized and supervised by an EBP mentor.

In this study there was a significant difference between the nurses who took part in evidence-based working groups and those who did not. Findings from a survey support the idea that mentorship in EBP facilitates the implementation of evidence-based care [58]. Polit and Beck [59] stressed the importance of education, administrative support, resources, and developing collaborations with potential mentors who can provide guidance and direction in the search for and appraisal of evidence.

Health political visions and goals require health staff to have competence in EBP and work in an evidence-based way, but implementing EBP among nurses in clinical practice is challenging. Although the use of questionnaires replied by individual nurses implies that we measure practice of EBP at the individual level, as nurses actually perform the components of EBP, we do not believe that practicing EBP is a purely individual responsibility. As nurses often say that they lack the autonomy to change practice [17,18], implementing EBP requires a whole system change implicating individuals, teams and the organization [60].

Effective change management plays a fundamental role facilitating an organizational environment that encourages EBP implementation [61-64]. The lead management plays a essential role in the technical and facilitative leadership, the organization’s policies, procedures, values, established habits, routines, financial and human resources and supervision of the clinical and non-clinical processes involved in EBP implementation.

### Methodological issues

One weakness of this cross-sectional study is the low response rate (52%). Possibly a higher response rate could have been achieved with more reminders. In our study, there was no difference in age or education between those who responded and those who did not respond. Nonetheless it is possible that those who answered were more positive towards EBP, and thus, caution must be used in generalizing the findings. There is a risk of receiving socially desirable responses where answers reflect an anticipated social norm, and retrospective self-reports about EBP beliefs and behaviour have been criticized for being biased [65].

The advantage of the questionnaires for the EBP Belief Scale and the EBP Implementation Scale are that they are standardised and have been used in a number of international studies. It is, however, difficult to achieve a high score on the EBP Implementation Scale. Even Wallen, Mitchell, Menyk et al.’s exclusive group of participants, who had been through an EBP mentorship program and who would ultimately mentor nurses in EBP, achieved an average score of less than half of highest possible score (40.9 of 72) [43]. So if an organization in their efforts to implement EBP focuses on a number of selected nurses becoming “experts” in EBP, and the rest of the staff being familiar with EBP, the average score of the EBP Implementation Scale for the whole staff is not likely to be high. To score highly all staff would have to carry out many activities related to EBP as part of their routine practice. This may be why the EBP Implementation Scale has mostly been used among samples who have a special interest in EBP or where the effect of EBP interventions are measured.

This survey was conducted at one particular hospital at a specific time. The nurses who work at this specialist hospital are known for being competent and at the forefront of their profession. Results from a similar study in other Norwegian hospitals may differ.

## Conclusion

Findings from this study add to the scientific arena of EBP that nurses are positive towards EBP, but to a lesser extent report practising EBP. There is a relationship between nurses’ beliefs towards EBP and the extent to which they report EBP implementation. Further, we see a link between each of the four subscales of the EBP Beliefs Scale and the EBP Implementation Scale, where beliefs related to knowledge are of the greatest importance for implementation.

Belief in the value of EBP was somewhat higher in the group that had learned about EBP than in the group which had not. In addition, there was a significant difference in those nurses who took part in evidence-based working groups. This may stress the importance of EBP- knowledge and skills, leadership and administrative support, financial and human resources, and developing collaborations with potential mentors.

While nurses can be taught how to use and perform the components of EBP, ongoing support in facilitating an evidence-based practice culture is necessary. It is therefore essential further explore how to best to organise the implementation of EBP in the health services effectively.

## Competing interests

The authors declare that they have no competing interests.

## Authors’ contributions

KS performed this study with supervision from NRO and MWN. MWN and NRO contributed to the conception and design of this study. KS collected the data. KS and BE contributed to the data analysis and interpretation of data. KS was responsible for the drafting of the manuscript. All authors revised it critically for important intellectual content and made a substantive contribution to revising the paper. All authors read and approved the final manuscript.

## Pre-publication history

The pre-publication history for this paper can be accessed here:

http://www.biomedcentral.com/1472-6955/13/8/prepub
